# Crystal structure of methyl 1-methyl-2-oxo­spiro­[indoline-3,2′-oxirane]-3′-carboxyl­ate

**DOI:** 10.1107/S2056989015006398

**Published:** 2015-04-02

**Authors:** M. P. Savithri, P. S. Yuvaraj, B. S. R. Reddy, R. Raja, A. SubbiahPandi

**Affiliations:** a Department of Physics, Queen Mary’s College (Autonomous), Chennai 600 004, India; b University of Madras, Industrial Chemistry Laboratory, Central Leather Research Institute, Adyar, Chennai 600 020, India; c Department of Physics, Presidency College (Autonomous), Chennai 600 005, India

**Keywords:** crystal structure, ester, spiro compound, indoline, oxirane, hydrogen bonding

## Abstract

In the title compound, C_12_H_11_NO_4_, the dihedral angle between the indole ring system (r.m.s. deviation = 0.019 Å) and the oxirane ring is 88.8 (2)°. The oxirane O atom and the bridging ester O atom are in an approximate syn conformation [O—C—C—O = −25.4 (3)°] In the crystal, inversion dimers linked by pair of C—H⋯O hydrogen bonds generate *R*_2_^2^(8) loops, where the C—H donor group forms part of the oxirane ring. A second C—H⋯O inter­action arising from one of the C—H groups of the benzene ring links the dimers into [001] double chains.

## Related literature   

For the bioactivity of indole derivatives, see: Di Fabio *et al.* (2007 ▸); Sharma & Tepe (2004 ▸). For a related structure, see: Savithri *et al.* (2015 ▸).
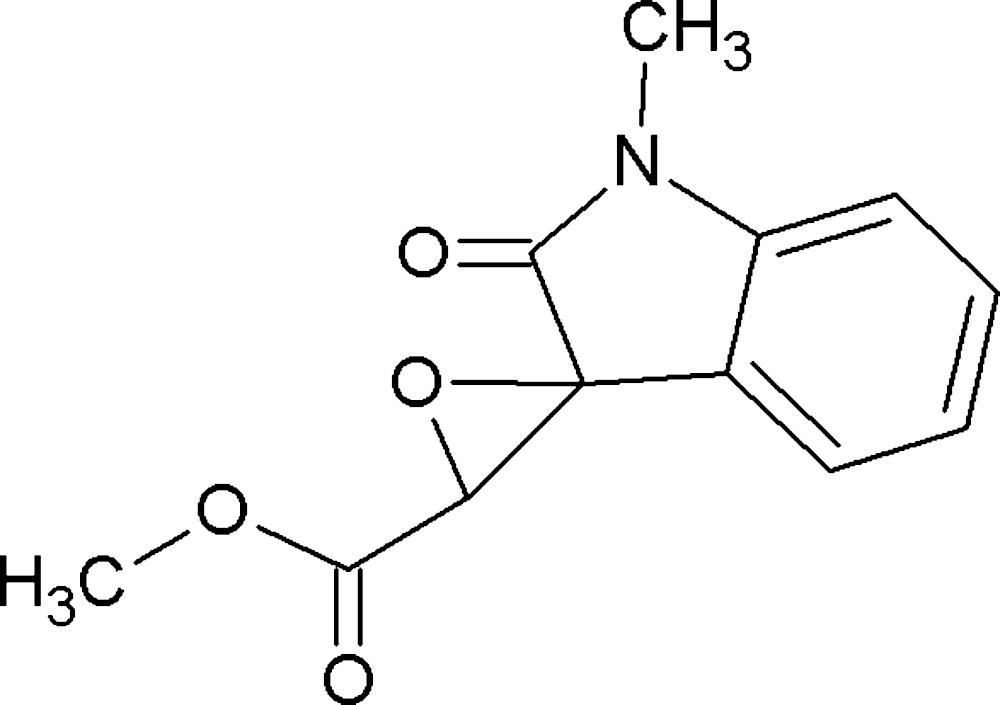


## Experimental   

### Crystal data  

C_12_H_11_NO_4_
*M* *_r_* = 233.22
Triclinic, 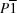

*a* = 7.1401 (4) Å
*b* = 8.7787 (4) Å
*c* = 9.0678 (4) Å
α = 91.517 (3)°
β = 104.227 (3)°
γ = 94.714 (3)°
*V* = 548.44 (5) Å^3^
*Z* = 2
Mo *K*α radiation
μ = 0.11 mm^−1^
*T* = 293 K
0.35 × 0.30 × 0.30 mm

### Data collection  

Bruker Kappa APEXII CCD diffractometer
Absorption correction: multi-scan (*SADABS*; Bruker, 2004 ▸) *T*_min_ = 0.963, *T*_max_ = 0.969
11311 measured reflections
1927 independent reflections
1480 reflections with *I* > 2σ(*I*)
*R*_int_ = 0.037

### Refinement  

*R*[*F* ^2^ > 2σ(*F* ^2^)] = 0.055
*wR*(*F* ^2^) = 0.173
*S* = 1.06
1927 reflections
158 parameters
H atoms treated by a mixture of independent and constrained refinement
Δρ_max_ = 0.30 e Å^−3^
Δρ_min_ = −0.27 e Å^−3^

### 

Data collection: *APEX2* (Bruker, 2004 ▸); cell refinement: *SAINT* (Bruker, 2004 ▸); data reduction: *SAINT*; program(s) used to solve structure: *SHELXS97* (Sheldrick, 2008 ▸); program(s) used to refine structure: *SHELXL97* (Sheldrick, 2008 ▸); molecular graphics: *ORTEP-3 for Windows* (Farrugia, 2012 ▸); software used to prepare material for publication: *SHELXL97* and *PLATON* (Spek, 2009 ▸).

## Supplementary Material

Crystal structure: contains datablock(s) global, I. DOI: 10.1107/S2056989015006398/hb7369sup1.cif

Structure factors: contains datablock(s) I. DOI: 10.1107/S2056989015006398/hb7369Isup2.hkl

Supporting information file. DOI: 10.1107/S2056989015006398/hb7369Isup3.cml

**Fig. 1.**
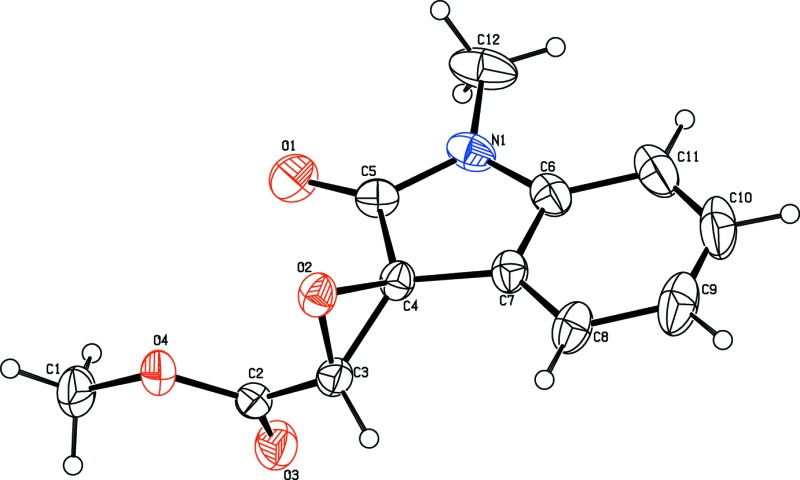
The molecular structure of the title compound with displacement ellipsoids drawn at the 30% probability level.

**Fig. 2.**
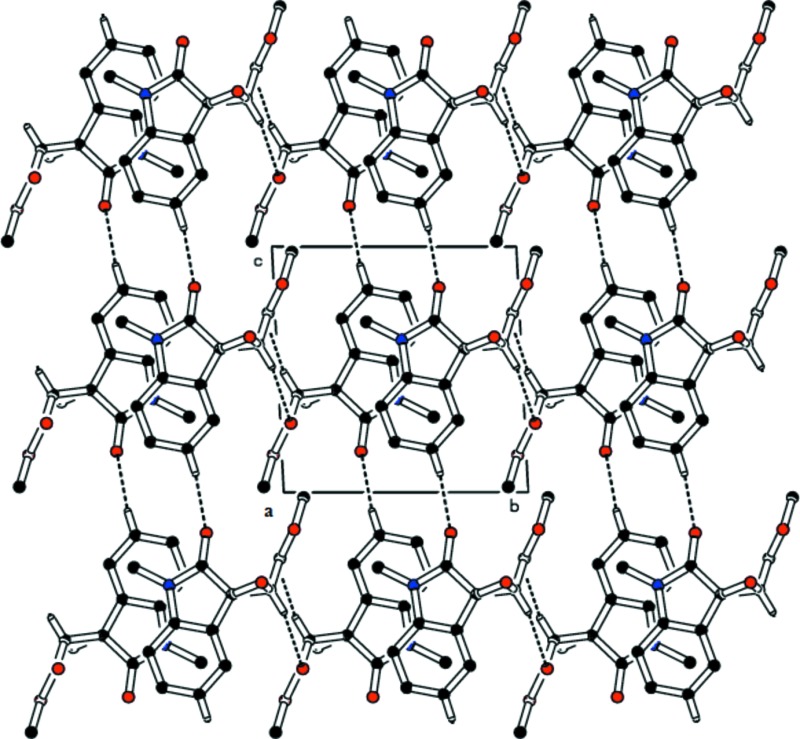
The molecular packing as viewed along the a axis. Dashed lines shows the C—H···O hydrogen bonds. H atoms not involved in hydrogen bonding have been omitted for clarity.

**Fig. 3.**
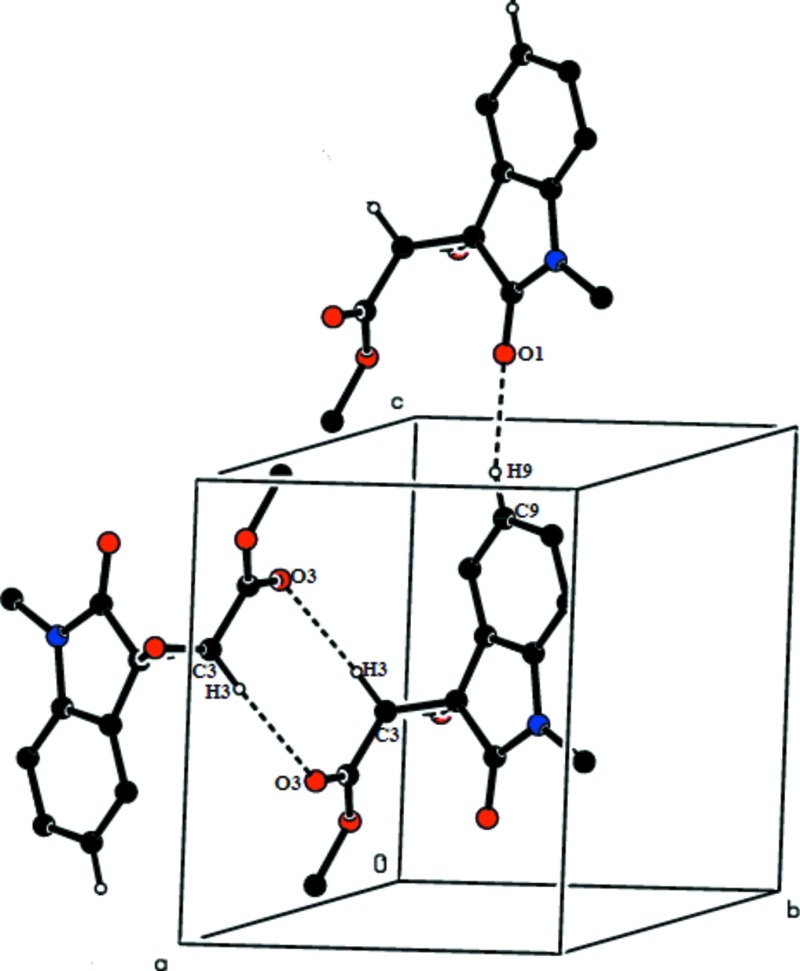
A partial view of the hydrogen-bond interactions C9—H9···O1 and C3—H3···O3 along a axis.

. DOI: 10.1107/S2056989015006398/hb7369fig1.tif
The mol­ecular structure of the title compound with displacement ellipsoids drawn at the 30% probability level.

a . DOI: 10.1107/S2056989015006398/hb7369fig2.tif
The mol­ecular packing as viewed along the *a* axis. Dashed lines shows the C—H⋯O hydrogen bonds. H atoms not involved in hydrogen bonding have been omitted for clarity.

a . DOI: 10.1107/S2056989015006398/hb7369fig3.tif
A partial view of the hydrogen-bond inter­actions C9—H9⋯O1 and C3—H3⋯O3 along *a* axis.

CCDC reference: 1056692

Additional supporting information:  crystallographic information; 3D view; checkCIF report

## Figures and Tables

**Table 1:** Hydrogen-bond geometry (, )

*D*H*A*	*D*H	H*A*	*D* *A*	*D*H*A*
C3H3O3^i^	0.95(3)	2.52(3)	3.414(3)	157(2)
C9H9O1^ii^	0.93	2.43	3.335(4)	163

Symmetry codes: (i) 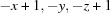; (ii) 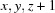.

## supplementary crystallographic information

### S1. Experimental

To a solution of the catalyst of diphenyl[(*R*)-2-pyrrolidinyl]methanol (0.15mmol) and trans-α-ylideneoxindoles 1 (0.5 mmol) in nHexane for HPLC grade (2.7ml) was added TBHP (5.5M in decane solution, 0.6mol) at room temperature (25°C). The resultant heterogeneous mixture was maintained under stirring until the reaction completion (TLC nHexane/EtOAc). After wards, the crude reaction mixture was purified by flash chromatography on silica gel (nHexane/EtOAc) to furnish the epoxy oxindoles trans-2 and cis-3. Colourless blocks were obtained by slow evaporation of a solution of the title compound in ethyl acetate at room temperature.

### S2. Refinement

All H atoms were fixed geometrically and allowed to ride on their parent C atoms, with C-H distances fixed in the range 0.93-0.98 Å with *U*_iso_(H) = 1.5*U*_eq_(C) for methyl H 1.2*U*_eq_(C) for other H atoms.

### Crystal data

**Table:**

C_12_H_11_NO_4_	*Z* = 2
*M**_r_* = 233.22	*F*(000) = 244
Triclinic, *P*1	*D*_x_ = 1.412 Mg m^−^^3^
Hall symbol: -P 1	Mo *K*α radiation, λ = 0.71073 Å
*a* = 7.1401 (4) Å	Cell parameters from 1927 reflections
*b* = 8.7787 (4) Å	θ = 2.3–25.0°
*c* = 9.0678 (4) Å	µ = 0.11 mm^−^^1^
α = 91.517 (3)°	*T* = 293 K
β = 104.227 (3)°	Block, colourless
γ = 94.714 (3)°	0.35 × 0.30 × 0.30 mm
*V* = 548.44 (5) Å^3^	

### Data collection

**Table:**

Bruker Kappa APEXII CCD diffractometer	1927 independent reflections
Radiation source: fine-focus sealed tube	1480 reflections with *I* > 2σ(*I*)
Graphite monochromator	*R*_int_ = 0.037
ω and φ scans	θ_max_ = 25.0°, θ_min_ = 2.3°
Absorption correction: multi-scan (*SADABS*; Bruker, 2004)	*h* = −8→8
*T*_min_ = 0.963, *T*_max_ = 0.969	*k* = −10→10
11311 measured reflections	*l* = −10→10

### Refinement

**Table:**

Refinement on *F*^2^	Primary atom site location: structure-invariant direct methods
Least-squares matrix: full	Secondary atom site location: difference Fourier map
*R*[*F*^2^ > 2σ(*F*^2^)] = 0.055	Hydrogen site location: inferred from neighbouring sites
*wR*(*F*^2^) = 0.173	H atoms treated by a mixture of independent and constrained refinement
*S* = 1.06	*w* = 1/[σ^2^(*F*_o_^2^) + (0.102*P*)^2^ + 0.172*P*] where *P* = (*F*_o_^2^ + 2*F*_c_^2^)/3
1927 reflections	(Δ/σ)_max_ < 0.001
158 parameters	Δρ_max_ = 0.30 e Å^−^^3^
0 restraints	Δρ_min_ = −0.27 e Å^−^^3^

### Special details

**Table:**

Geometry. All esds (except the esd in the dihedral angle between two l.s. planes) are estimated using the full covariance matrix. The cell esds are taken into account individually in the estimation of esds in distances, angles and torsion angles; correlations between esds in cell parameters are only used when they are defined by crystal symmetry. An approximate (isotropic) treatment of cell esds is used for estimating esds involving l.s. planes.
Refinement. Refinement of F^2^ against ALL reflections. The weighted R-factor wR and goodness of fit S are based on F^2^, conventional R-factors R are based on F, with F set to zero for negative F^2^. The threshold expression of F^2^ > 2sigma(F^2^) is used only for calculating R-factors(gt) etc. and is not relevant to the choice of reflections for refinement. R-factors based on F^2^ are statistically about twice as large as those based on F, and R- factors based on ALL data will be even larger.

### Fractional atomic coordinates and isotropic or equivalent isotropic displacement parameters (Å^2^)

**Table:**

	*x*	*y*	*z*	*U*_iso_*/*U*_eq_	
O2	0.0254 (2)	0.10821 (18)	0.36778 (18)	0.0462 (5)	
O4	0.1745 (3)	−0.03538 (19)	0.15149 (19)	0.0536 (5)	
O3	0.4764 (3)	0.0432 (2)	0.2809 (2)	0.0632 (6)	
O1	0.1739 (3)	0.3246 (2)	0.1663 (2)	0.0643 (6)	
N1	0.2372 (3)	0.4866 (2)	0.3794 (3)	0.0504 (6)	
C7	0.2040 (3)	0.3195 (3)	0.5623 (3)	0.0425 (6)	
C2	0.3047 (4)	0.0282 (2)	0.2698 (3)	0.0429 (6)	
C4	0.1736 (3)	0.2320 (2)	0.4166 (2)	0.0379 (5)	
C3	0.2223 (4)	0.0750 (3)	0.3974 (3)	0.0428 (6)	
C6	0.2456 (3)	0.4711 (3)	0.5343 (3)	0.0486 (6)	
C5	0.1928 (3)	0.3492 (3)	0.3012 (3)	0.0438 (6)	
C8	0.2075 (4)	0.2738 (4)	0.7065 (3)	0.0566 (7)	
H8	0.1811	0.1715	0.7247	0.068*	
C11	0.2901 (4)	0.5825 (4)	0.6509 (4)	0.0717 (10)	
H11	0.3174	0.6849	0.6334	0.086*	
C1	0.2456 (5)	−0.0803 (4)	0.0221 (3)	0.0692 (9)	
H1A	0.1393	−0.1255	−0.0577	0.104*	
H1B	0.3391	−0.1534	0.0521	0.104*	
H1C	0.3056	0.0081	−0.0141	0.104*	
C12	0.2681 (5)	0.6277 (3)	0.3066 (5)	0.0774 (10)	
H12A	0.2979	0.7114	0.3813	0.116*	
H12B	0.1528	0.6440	0.2303	0.116*	
H12C	0.3742	0.6214	0.2597	0.116*	
C10	0.2921 (5)	0.5344 (5)	0.7951 (4)	0.0845 (12)	
H10	0.3223	0.6068	0.8761	0.101*	
C9	0.2516 (5)	0.3848 (5)	0.8237 (4)	0.0796 (11)	
H9	0.2537	0.3576	0.9225	0.096*	
H3	0.273 (4)	0.024 (3)	0.488 (3)	0.052 (7)*	

### Atomic displacement parameters (Å^2^)

**Table:**

	*U*^11^	*U*^22^	*U*^33^	*U*^12^	*U*^13^	*U*^23^
O2	0.0454 (10)	0.0440 (9)	0.0450 (9)	−0.0086 (7)	0.0076 (7)	0.0005 (7)
O4	0.0565 (11)	0.0523 (11)	0.0456 (10)	0.0033 (8)	0.0026 (8)	−0.0115 (8)
O3	0.0524 (13)	0.0730 (13)	0.0597 (12)	0.0065 (9)	0.0068 (9)	−0.0112 (9)
O1	0.0859 (15)	0.0673 (12)	0.0455 (11)	0.0096 (10)	0.0247 (10)	0.0167 (9)
N1	0.0464 (12)	0.0339 (11)	0.0738 (15)	0.0021 (8)	0.0206 (10)	0.0087 (9)
C7	0.0352 (12)	0.0495 (14)	0.0418 (13)	0.0004 (10)	0.0099 (9)	−0.0061 (10)
C2	0.0477 (15)	0.0335 (12)	0.0425 (13)	0.0041 (10)	0.0020 (10)	0.0010 (9)
C4	0.0380 (12)	0.0387 (12)	0.0351 (11)	−0.0034 (9)	0.0078 (9)	0.0011 (9)
C3	0.0477 (14)	0.0373 (12)	0.0382 (13)	−0.0010 (10)	0.0024 (10)	0.0032 (10)
C6	0.0333 (13)	0.0470 (14)	0.0650 (17)	0.0033 (10)	0.0125 (11)	−0.0104 (11)
C5	0.0425 (13)	0.0441 (13)	0.0476 (14)	0.0047 (10)	0.0154 (10)	0.0087 (10)
C8	0.0495 (15)	0.0805 (19)	0.0392 (14)	−0.0009 (13)	0.0128 (11)	−0.0032 (12)
C11	0.0491 (17)	0.0595 (18)	0.100 (3)	0.0038 (13)	0.0116 (16)	−0.0328 (17)
C1	0.077 (2)	0.082 (2)	0.0455 (15)	0.0188 (16)	0.0083 (13)	−0.0170 (14)
C12	0.0574 (18)	0.0453 (16)	0.137 (3)	0.0067 (13)	0.0348 (18)	0.0312 (17)
C10	0.061 (2)	0.110 (3)	0.074 (2)	0.0127 (19)	0.0073 (16)	−0.052 (2)
C9	0.062 (2)	0.128 (3)	0.0476 (17)	0.0067 (19)	0.0138 (14)	−0.0251 (18)

### Geometric parameters (Å, º)

**Table:**

O2—C3	1.421 (3)	C3—H3	0.95 (3)
O2—C4	1.433 (3)	C6—C11	1.380 (4)
O4—C2	1.311 (3)	C8—C9	1.382 (4)
O4—C1	1.446 (3)	C8—H8	0.9300
O3—C2	1.201 (3)	C11—C10	1.382 (5)
O1—C5	1.209 (3)	C11—H11	0.9300
N1—C5	1.356 (3)	C1—H1A	0.9600
N1—C6	1.402 (3)	C1—H1B	0.9600
N1—C12	1.446 (3)	C1—H1C	0.9600
C7—C8	1.373 (3)	C12—H12A	0.9600
C7—C6	1.384 (4)	C12—H12B	0.9600
C7—C4	1.470 (3)	C12—H12C	0.9600
C2—C3	1.484 (4)	C10—C9	1.367 (5)
C4—C3	1.466 (3)	C10—H10	0.9300
C4—C5	1.509 (3)	C9—H9	0.9300
			
C3—O2—C4	61.80 (14)	O1—C5—C4	126.6 (2)
C2—O4—C1	116.2 (2)	N1—C5—C4	106.3 (2)
C5—N1—C6	111.2 (2)	C7—C8—C9	117.8 (3)
C5—N1—C12	122.4 (3)	C7—C8—H8	121.1
C6—N1—C12	126.4 (2)	C9—C8—H8	121.1
C8—C7—C6	121.6 (2)	C6—C11—C10	116.7 (3)
C8—C7—C4	131.6 (2)	C6—C11—H11	121.7
C6—C7—C4	106.7 (2)	C10—C11—H11	121.7
O3—C2—O4	125.0 (2)	O4—C1—H1A	109.5
O3—C2—C3	121.3 (2)	O4—C1—H1B	109.5
O4—C2—C3	113.7 (2)	H1A—C1—H1B	109.5
O2—C4—C3	58.71 (14)	O4—C1—H1C	109.5
O2—C4—C7	123.07 (19)	H1A—C1—H1C	109.5
C3—C4—C7	125.9 (2)	H1B—C1—H1C	109.5
O2—C4—C5	116.78 (18)	N1—C12—H12A	109.5
C3—C4—C5	121.0 (2)	N1—C12—H12B	109.5
C7—C4—C5	105.51 (19)	H12A—C12—H12B	109.5
O2—C3—C4	59.48 (14)	N1—C12—H12C	109.5
O2—C3—C2	119.95 (19)	H12A—C12—H12C	109.5
C4—C3—C2	121.3 (2)	H12B—C12—H12C	109.5
O2—C3—H3	117.1 (15)	C9—C10—C11	122.8 (3)
C4—C3—H3	116.4 (16)	C9—C10—H10	118.6
C2—C3—H3	112.8 (15)	C11—C10—H10	118.6
C11—C6—C7	120.9 (3)	C10—C9—C8	120.3 (3)
C11—C6—N1	128.8 (3)	C10—C9—H9	119.9
C7—C6—N1	110.3 (2)	C8—C9—H9	119.9
O1—C5—N1	127.2 (2)		
			
C1—O4—C2—O3	−4.4 (4)	C4—C7—C6—N1	2.0 (3)
C1—O4—C2—C3	178.6 (2)	C5—N1—C6—C11	178.4 (2)
C3—O2—C4—C7	−115.0 (2)	C12—N1—C6—C11	−2.3 (4)
C3—O2—C4—C5	111.7 (2)	C5—N1—C6—C7	−0.6 (3)
C8—C7—C4—O2	44.0 (4)	C12—N1—C6—C7	178.7 (2)
C6—C7—C4—O2	−140.1 (2)	C6—N1—C5—O1	179.6 (2)
C8—C7—C4—C3	−28.9 (4)	C12—N1—C5—O1	0.2 (4)
C6—C7—C4—C3	147.0 (2)	C6—N1—C5—C4	−1.0 (3)
C8—C7—C4—C5	−178.4 (2)	C12—N1—C5—C4	179.7 (2)
C6—C7—C4—C5	−2.5 (2)	O2—C4—C5—O1	−37.7 (3)
C4—O2—C3—C2	−110.9 (2)	C3—C4—C5—O1	30.3 (4)
C7—C4—C3—O2	110.4 (2)	C7—C4—C5—O1	−178.4 (2)
C5—C4—C3—O2	−104.4 (2)	O2—C4—C5—N1	142.9 (2)
O2—C4—C3—C2	108.6 (2)	C3—C4—C5—N1	−149.1 (2)
C7—C4—C3—C2	−141.0 (2)	C7—C4—C5—N1	2.2 (2)
C5—C4—C3—C2	4.2 (3)	C6—C7—C8—C9	0.9 (4)
O3—C2—C3—O2	157.5 (2)	C4—C7—C8—C9	176.2 (3)
O4—C2—C3—O2	−25.4 (3)	C7—C6—C11—C10	0.5 (4)
O3—C2—C3—C4	87.1 (3)	N1—C6—C11—C10	−178.5 (2)
O4—C2—C3—C4	−95.8 (2)	C6—C11—C10—C9	−0.3 (5)
C8—C7—C6—C11	−0.8 (4)	C11—C10—C9—C8	0.5 (5)
C4—C7—C6—C11	−177.1 (2)	C7—C8—C9—C10	−0.7 (4)
C8—C7—C6—N1	178.4 (2)		

### Hydrogen-bond geometry (Å, º)

**Table:**

*D*—H···*A*	*D*—H	H···*A*	*D*···*A*	*D*—H···*A*
C3—H3···O3^i^	0.95 (3)	2.52 (3)	3.414 (3)	157 (2)
C9—H9···O1^ii^	0.93	2.43	3.335 (4)	163

Symmetry codes: (i) −*x*+1, −*y*, −*z*+1; (ii) *x*, *y*, *z*+1.
