# Supplementary figures and images for: Crystal structure of methyl 1-methyl-2-oxo­spiro­[indoline-3,2′-oxirane]-3′-carboxyl­ate
Source: Acta Crystallogr E Crystallogr Commun. 2015 Apr 2;71(Pt 5):o274–5. doi: 10.1107/S2056989015006398 (PMC4420098; doi:10.1107/S2056989015006398)

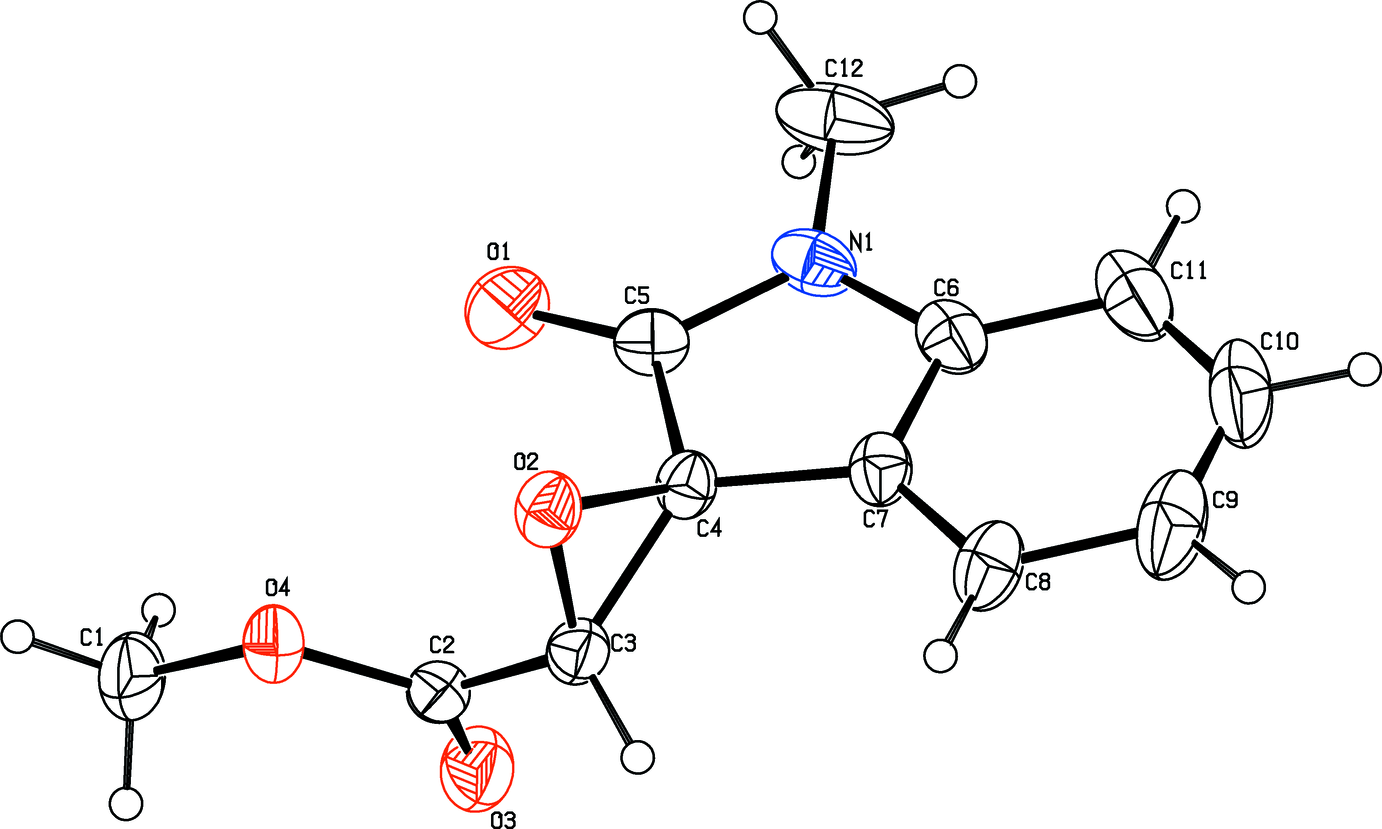

Supplement: Supplementary file 4 [file e-71-0o274-fig1.tif]

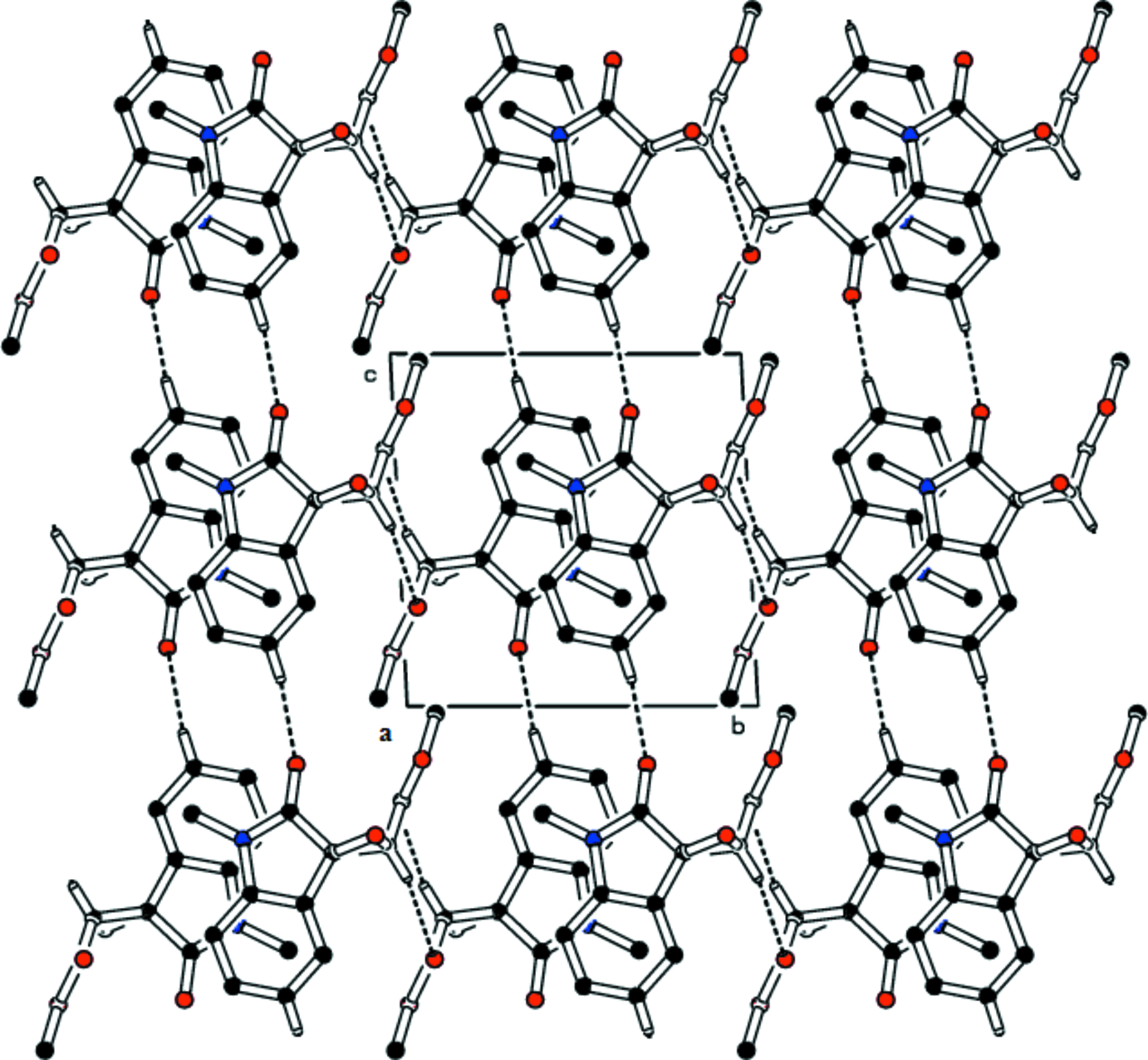

Supplement: Supplementary file 5 [file e-71-0o274-fig2.tif]

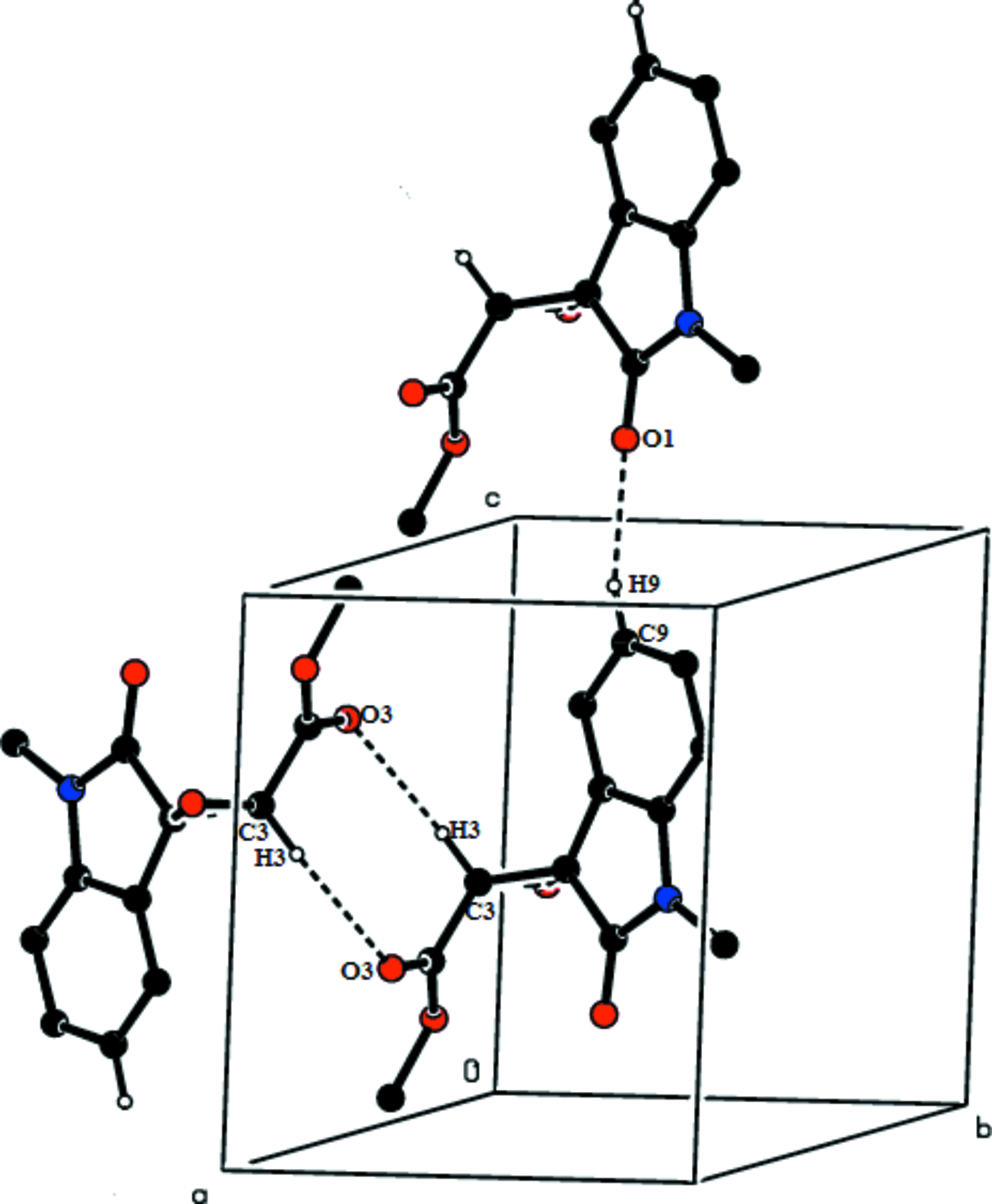

Supplement: Supplementary file 6 [file e-71-0o274-fig3.tif]
